# *In Vitro* Grown Pollen Tubes of *Nicotiana alata* Actively Synthesise a Fucosylated Xyloglucan

**DOI:** 10.1371/journal.pone.0077140

**Published:** 2013-10-08

**Authors:** Edwin R. Lampugnani, Isabel E. Moller, Andrew Cassin, Daniel F. Jones, Poh Ling Koh, Sunil Ratnayake, Cherie T. Beahan, Sarah M. Wilson, Antony Bacic, Ed Newbigin

**Affiliations:** 1 School of Botany, University of Melbourne, Melbourne, Victoria, Australia; 2 Australian Research Council Centre of Excellence in Plant Cell Walls, School of Botany, University of Melbourne, Melbourne, Victoria, Australia; 3 Bio21 Institute for Molecular Science & Biotechnology, University of Melbourne, Victoria, Australia; 4 Department of Botany, La Trobe University, Bundoora, Victoria, Australia; Wuhan University, China

## Abstract

*Nicotiana alata* pollen tubes are a widely used model for studies of polarized tip growth and cell wall synthesis in plants. To better understand these processes, RNA-Seq and *de novo* assembly methods were used to produce a transcriptome of *N. alata* pollen grains. Notable in the reconstructed transcriptome were sequences encoding proteins that are involved in the synthesis and remodelling of xyloglucan, a cell wall polysaccharide previously not thought to be deposited in *Nicotiana* pollen tube walls. Expression of several xyloglucan-related genes in actively growing pollen tubes was confirmed and xyloglucan epitopes were detected in the wall with carbohydrate-specific antibodies: the major xyloglucan oligosaccharides found in *N. alata* pollen grains and tubes were fucosylated, an unusual structure for the Solanaceae, the family to which *Nicotiana* belongs. Finally, carbohydrate linkages consistent with xyloglucan were identified chemically in the walls of *N. alata* pollen grains and pollen tubes grown in culture. The presence of a fucosylated xyloglucan in *Nicotiana* pollen tube walls was thus confirmed. The consequences of this discovery to models of pollen tube growth dynamics and more generally to polarised tip-growing cells in plants are discussed.

## Introduction

*Nicotiana* pollen tubes (*N. tabacum* and *N. alata*) are a widely used and a well-characterised system for studying polar cell growth and cell wall synthesis in plants [1-3]. *Nicotiana* pollen tubes elongate rapidly in a strictly polar manner via the tip-focused fusion of polysaccharide-laden secretory vesicles and deposition of newly synthesized material into the nascent primary wall that forms at the tube tip or apex. Deposition of more polysaccharide at some distance behind the apex reinforces the primary wall and produces a thicker inner secondary wall [4]. At intervals along distal regions of the pollen tube shank are transverse callose-containing cross-walls called plugs that act to seal the cytoplasmic living portion of the pollen tube, containing the sperm cells, off from spent portions of the tube further back towards the grain. Given this structure, it is apparent that forming the pollen tube cell wall requires precise control over the spatial distribution of the various glycosyl synthases and transferases needed to make the limited number of polysaccharides that are found in the wall, which is predominantly composed of the (1,3)-β-D-glucan callose and lesser amounts of cellulose, a neutral pectic arabinan and acidic pectins [5,6]. Of these polysaccharides, only callose and cellulose have been associated with candidate genes and enzymes in pollen tubes [7,8].

To identify additional genes involved in wall polysaccharide biosynthesis in *Nicotiana* pollen tubes, an RNA-Seq approach associated with *de novo* transcriptome reconstruction [9] was used to prepare a draft *N. alata* pollen grain transcriptome. We were surprised to discover the transcriptome contained several contigs derived from cDNAs related to the synthesis and remodelling of xyloglucan (XyG), a polysaccharide that was not thought to be deposited in *Nicotiana* pollen tube walls [6]. Using the transcriptome we identified full-length cDNAs for most of the glycosyl synthases and transferases needed to assemble and remodel XyG, and subsequently showed that *Nicotiana* pollen tubes actively synthesise and deposit in their wall a highly branched XyG that is substituted with fucose: neither the degree of branching nor the presence of fucose is typical of the XyG structures previously reported for solanaceous plants like tobacco (e.g., see 10). Discovering this polysaccharide requires a reassessment of the contribution a network of cellulose microfibrils and interacting XyGs may make to the mechanical properties of *Nicotiana* pollen tube walls.

## Materials and Methods

### Plant materials

*Nicotiana alata* plants (self-incompatibility genotype S_2_S_3_) were grown in soil in a glasshouse as previously described [11]. Pollen was collected and stored at -80°C until used. Growth medium and culture conditions for pollen were as described by Li et al. [12].

### *de novo* transcriptome assembly of *N. alata* pollen grains

Total RNA from *N. alata* pollen grains was extracted as described [7] and sent to the Australian Genome Research Facility (service provider; Brisbane, Australia) for mRNA-SEQ library preparation and sequencing. The raw 75 bp reads produced by an Illumina GA-II sequencer from pre-processed using DynamicTrim v 1.9 [13] at a non-stringent filtering PHRED value of 4 (i.e. only reads with a PHRED score >4 were used) to remove very low quality reads and *de novo* assembled using Trinity v r2011-08-20 [14]. To calculate the RPKM (Reads Per Kilobase of exon model per Million mapped reads) expression value for each contig, the original (unprocessed) reads were mapped onto the assembly generated by Trinity using the proprietary read mapping algorithm of CLC Genomics Workbench (http://www.clcbio.com). The 75 bp reads were mapped to the draft *N. benthamiana* genome version 0.4.2 [15] using Bowtie version 2.0.6 [16].

### Molecular biology and bioinformatics

Total RNA from *N. alata* leaf, pollen and pollen tubes was extracted using a RNeasy Plant Mini Kit (Qiagen) and DNA contamination removed by treating the RNA (5 µg) with 2 U of DNase I (Life Technologies). First-strand cDNA synthesis was carried out using an oligo dT17 primer and 200 units of Superscript III (Life Technologies) with the further addition of 40 units of RNaseOUT Recombinant RNase Inhibitor (Life Technologies). Sequences of the gene-specific primers used in this experiment are shown in Table S3. PCR was performed with 1 µl of cDNA template in a final volume of 25 µl of 1× PCR buffer containing template DNA, 0.6 µM of each primer, 0.4 mM dNTPs, 1.5 mM MgCl_2_ and 2 units of Taq polymerase (Scientifix) on a GeneAmp 2700 thermal cycler (Applied Biosystems). Cycling conditions were as follows: 95°C 2 min, then 35 cycles (95°C, 30 s; 55°C, 30 s; 72°C, 25 s). PCR products were purified using the QIAquick PCR purification kit (Qiagen) and sequenced directly using the service provided by the Australian Genome Research Facility (Melbourne, Australia). 

Sequences alignments were generated with the ClustalW program [17] using Geneious Pro 5.6.3 software (Biomatters) and the resulting alignments were then verified by eye. The Basic Local Alignment Search Tool (BLAST; [18]) was used to query NCBI databases for highly similar sequences. 

Sequences of the contigs described in the text have been deposited in DDBJ with accession numbers AB844117 - AB844170. These numbers are listed in Tables S2 and S3.

### Microscopy

For immuno-fluorescence detection of cell wall polysaccharides, *Nicotiana* pollen tubes were grown in culture for 4 and 16 hr and fixed in 4% paraformaldehyde. Tissue was pre-incubated in blocking buffer (3% milk powder in 1× phosphate-buffered saline (PBS); 1× PBS is 37 mM NaCl, 10 mM PO_4_ and 2.7 mM KCl, pH 7.4) for 1 hr to prevent non-specific antibody binding. Monoclonal antibodies were diluted in blocking buffer as follows: LM15 (PlantProbes, 1/10 dilution Rat IgG2c), CCRC-M1 (CarboSource, 1/200 dilution IgG1), Callose (Biosupplies Australia; 1/100 dilution IgG1), and then incubated for 2.5 hr at 4°C, washed in blocking buffer (×2), before overnight incubation in either a 1:100 dilution of anti-mouse Alexa fluor 488 (Life Technologies). Samples were washed in PBS twice before being mounted on slides and counter-stained with N-(3-triethylammoniumpropyl)-4-(6-(4-(diethylamino)phenyl)hexatrienyl) pyridinium dibromide (FM4-64; Life Technologies) at 10 μg/ml. Slides were viewed on an inverted Leica SP2 confocal microscope using a 63× PL Apo BL oil objective (n.a. 1.4). A 488 nm laser line, attenuated to 20%, was used to excite both the Alexa Fluor 488 and FM4-64. Emissions were detected simultaneously between 498-530 nm for Alexa fluor 488 and 650-800 nm for FM4-64. Photo-detectors were set at 70 and 30, respectively, offset by -5. All settings were held constant. Images were collected using the average of four optical slices and z-stacks were taken with successive 0.25 μm scans.

Pontamine Fast Scarlet 4B (S4B) was a gift from Prof Chris Somerville (Energy Biosciences Institute, University of California at Berkeley). Pollen tubes were suspended in a 0.01% (w/v) solution of S4B in 1× PBS or 0.003% Aniline Blue fluorochrome (Biosupplies Australia) in water for 30 min, washed twice in 10% glycerol before mounting on slides for imaging with a confocal microscope as described above except that a 405 nm laser line, attenuated to 20%, was used to excite the Aniline Blue fluorochrome and emissions were detected between 415-550 nm and S4B samples were imaged using a 543 nm laser line, attenuated to 20%, and emissions were detected between 550-800 nm. 

For detection of XyG by immuno-electron microscopy, pollen tubes were processed by the high-pressure freezing method described in Brownfield et al. [19]. Thin sections were incubated in a 1:50 dilution of LM15 in PBS containing 1% w/v BSA for 1 hr at room temperature and then overnight at 4°C. Grids were washed in PBS and then incubated in a 1:20 dilution of goat anti-rat secondary antibody conjugated to 18-nm gold particles (Jackson ImmunoResearch). Sections were washed, post-stained and viewed by transmission electron microscopy as described [20].

### Preparation of cell walls and analysis of XyG oligosaccharides

Fingerprinting of XyG oligosaccharides was done as described by Lerouxel et al. [21]. *Nicotiana* pollen grain and tube cell wall material (10 mg) was boiled for 10 min in sodium acetate buffer (50 mM, pH 5) and incubated with agitation for 24 hr at 37°C with 0.4 units/mg cellulase (endo-1,4-β-D-glucanase; Megazyme, Ireland) in NaOAc buffer (50 mM, pH 5). The enzyme was deactivated by boiling samples for 15 min, any remaining insoluble material was removed by centrifugation and then following the addition of ethanol (4 vols) the XyG oligosaccharides was isolated from the supernatant and vacuum dried.

Samples were mixed in a 1:1 ratio with matrix containing 50% dihydro benzoic acid (DHB) / 50% acetonitrile containing 0.1% formic acid and analysed using a MicroFlex MALDI-TOF instrument (Bruker Daltonics, Germany). The spectra were acquired using Flex Control software Version 3.3 in positive ion reflector mode using an acceleration of 19.0 kV at ion source 1 and 16.3 kV at ion source 2 with a delay time of 200 ns and greater than 70% laser power. The spectra were calibrated using Bruker Daltonics Peptide Calibration Standard II. 

Electrospray ionization–tandem mass spectrometry (ESI-MS^n^) analysis was performed for the predominant quasi-molecular ions (m/z 1085, 1435 and 1639) using an Agilent LC/MSD ion-trap XCT plus MS (Agilent, Palo Alto, CA). Samples were infused with acetonitrile (50%) through an Agilent HPLC calibration chip into the source at a flow rate of 0.5 µl / min using an automated syringe pump. The ESI source was operated at a voltage of 1950V, and the capillary heater was set to 300 °C. All the experiments were performed in the positive-ion mode.

## Results

### Identification of XyG-related genes in *Nicotiana* pollen grains and tubes

RNA-Seq analysis of *Nicotiana* pollen grain poly (A+) RNA generated more than a 7.6 million 75 bp single-end reads (Table S1). A *de novo* assembly produced a transcriptome containing 11,049 contigs of 200 bp or more in length. The total length of the reconstructed transcriptome was 5,828,264 bp; the largest contig in the transcriptome was 6,983 bp, the average contig length was 528 bp and the median length was 341 bp (Table S1). Using Bowtie [16] more than 90% of the reads in the assembly could be aligned to the recently published *N. benthamiana* genome (Table S1; [15]). 

To determine the representation of known *Nicotiana* sequences in the transcriptome, 56 pollen-expressed cDNAs (17 from *N. alata* and 39 from *N. tabacum*) were used to query the database (Table S2). Of the 56 cDNAs, 38 (9 from *N. alata* and 29 from *N. tabacum*) had a >90% pairwise identity match over most of their length to at least one contig in the assembly. Although this suggests that the transcriptome contained approximately 70% of the known transcripts, this is probably an underestimate as some of the genes in the list, such as *N. alata CELLULOSE SYNTHASE1* (*NaCESA1*), are known to be expressed at very low levels in pollen grains [7]. Consistent with this, no unique reads for *NaCESA1* were found in the RNA-Seq data. Of the 8 *N. alata* cDNAs with no clear match in the assembly, 6 are from a family of F-box protein-encoding genes that are associated with the *N. alata* self-incompatibility response (*DD1-10*; [22]). Although the RNA-Seq data contained reads for all the missing *DD* cDNAs, the contigs assembled from these reads were excluded from the transcriptome because none was over 200 bp in length. RPKM values, a measure of relative expression level between contigs, for the 38 known pollen transcripts ranged from 17 to 37,610, demonstrating that contigs >200 bp in length were successfully assembled for transcripts with expression differences ranging over three orders of magnitude. The transcriptome included contigs matching previously studied pollen-expressed genes from *N. alata* related to cell wall synthesis such as *NaGSL1* (*N. alata GLUCAN SYNTHASE-LIKE 1*), which encodes the putative callose synthase [8], and *NaCSLD1* (*N. alata CELLULOSE SYNTHASE-LIKE D1*), which encodes the putative cellulose synthase [7]. 

Searching the transcriptome for cell-wall synthesis related genes identified several contigs that were derived from genes involved in XyG biosynthesis and remodelling. To identify the full complement of XyG-related genes the pollen grain transcriptome was queried using the sequences of relevant *Arabidopsis* genes [23,24]. The longest open reading frame from each *Nicotiana* contig was then used to search the non-redundant protein sequence database at TAIR (www.arabidopsis.org) and the top result used as the match for that contig. 

**Figure 1:**
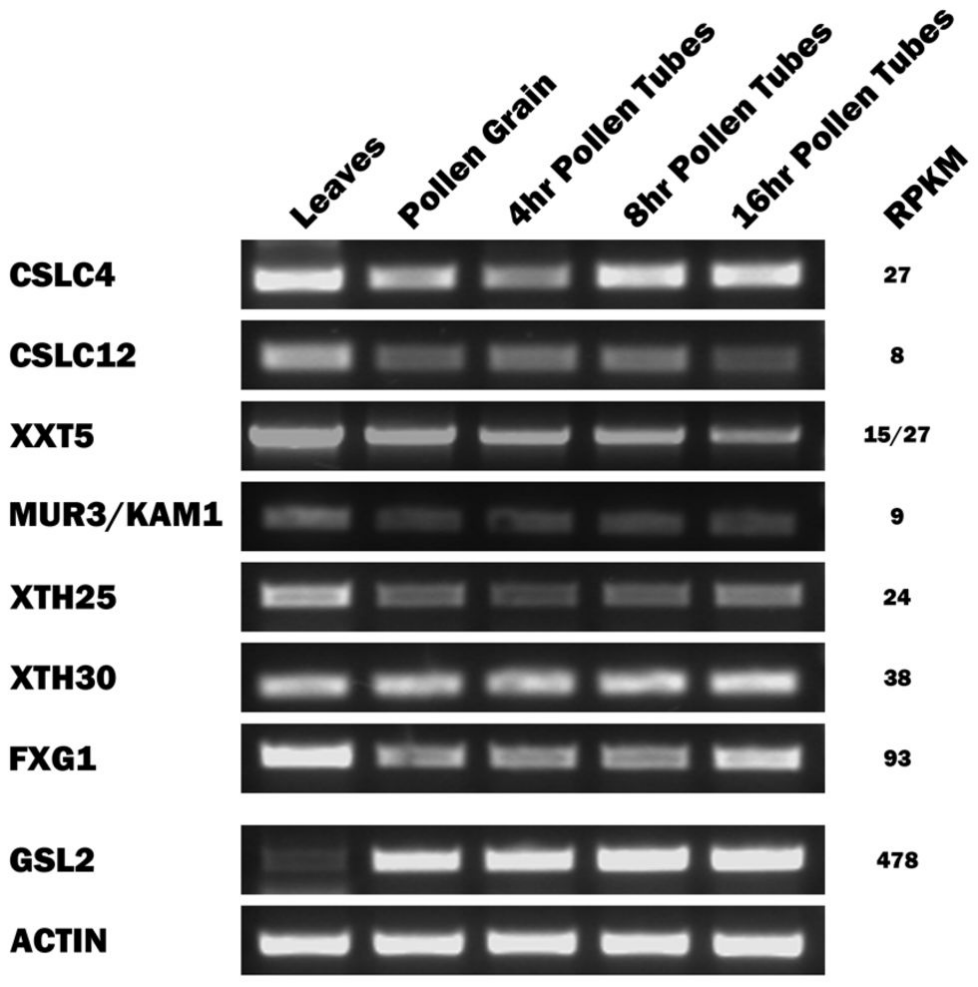
Expression profiles of XyG-related genes in various *N. alata* tissues. RT–PCR was carried out using the indicated cDNA template and primers (see Table S3) specific for each of the XyG-related gene listed in Table 1. RT-PCR for each template using actin-specific primers (positive control) is also shown.

Table 1 shows the 8 XyG-related contigs that were identified in the pollen grain transcriptome and their best *Arabidopsis* matches. All of the contigs could be amplified from pollen grain cDNA (Figure 1). As measured by RPKM values, the 8 XyG-related genes were expressed at levels that were <15% (and in most instances <6%) of the level of *NaGSL1* (compare Table 1 and Table S2). 

**Table 1:** Xyloglucan-related genes identified in the *N. alata* pollen grain transcriptome.

**Query**		**Number of hits**	**Contig RPKM value**	**Top reciprocal BLAST hit**	
**Locus**	**Predicted function**			**Locus**	**Abbreviation**
At2g03220	(1,2)-α-L-fucosyltransferase	0	-	At2g03220	*At*FUT1
At3g28180	(1-4)-β-D-glucansynthase	1	27	At3g28180	*At*CSLC4
At4g07960	(1-4)-β-D-glucansynthase	1	8	At4g07960	*At*CSLC12
At1g74380	(1-6)-α-D-xylosyltransferase	2	15/27	At1g74380	*At*XXT5
At2g20370	(1-2)-β-galactosyltransferase	1	9	At2g20370	*At*MUR3/*At*KAM1
At5g57550	endo-(1-4)-β-D-glucanase	1	24	At5g57550	*At*XTH25
At1g32170	endo-(1-4)-β-D-glucanase	1	38	At1g32170	*At*XTH30
At1g67830	(1-2)-α-L-fucosidase	1	93	At1g67830	*At*FXG1
At5g63810	β-galactosidase	1	3,994	AT5G63800	BGAL6/MUM3

Translated peptide sequences of previously characterised XyG biosynthesis genes from *Arabidopsis* were used to search for related sequences in the *N. alata* pollen transcriptome. RPKM is a measure of the level of expression of a contig.

The XyG in most flowering plants has a (1,4)-β-glucan backbone decorated at the C(O)6 position of the Glc with α-xylopyranosyl residues that can be extended by subsequent additions of a (1,2)-β-galactopyranosyl unit, often terminating with a (1,2)-α-fucopyranosyl residue. Synthesis of this structure requires at a minimum one (1,4)-β-glucan synthase (synthesising the glucan backbone), a (1,6)-α-xylosyltransferase, a (1,2)-β-galactosyltransferase and a (1,2)-α-fucosyltransferase (synthesising the branches). With the exception of the fucosyltransferase, candidate genes encoding all the other biosynthetic activities were found (Table 1). Specifically there were two separate Cellulose Synthase-Like subfamily C (CSLC) contigs, with the best *Arabidopsis* match for one of these being *AtCSLC4*, which encodes the (1-4)-β-glucan synthase that makes the XyG backbone [25]. Additionally, there were two non-overlapping contigs that had best *Arabidopsis* matches to *AtXXT5*, a gene for a XyG (1,6)-α-xylosyltransferase shown to form a complex with *At*CSLC4 [26,27]. Finally, there was a contig coding for a (1,2)-β-galactosyltransferase that was a best match in *Arabidopsis* to MUR3, which Madson et al. [28] showed has XyG galactosyltransferase activity.

In addition to synthase/transferase contigs, the pollen grain transcriptome also contained two contigs for XYLOGLUCAN endo-TRANSGLYCOSYLASE/HYDROLASE (XTH) proteins that are associated with XyG remodeling (Table 1). XTH proteins are associated with two distinct catalytic activities: a XyG endo-transglycolase (XET) activity that brings about the cleavage and religation of XyG chains; and a XyG-specific endo-hydrolase (XEH) activity that hydrolytically cleaves and thereby shortens XyG chains [29]. The best *Arabidopsis* matches for the two pollen grain contigs are to *At*XTH25, which falls into XTH group I/II, and *At*XTH30, which is a group III-B XTH (classification as described in [29]). XET activity is the only demonstrated activity for proteins in groups I/II and III-B.

Other contigs found in the transcriptome and potentially associated with XyG metabolism encoded an α-fucosidase and a β-galactosidase (Table 1). The α-fucosidase was related to *At*FXG1, an apoplastic enzyme from *Arabidopsis* able to remove terminal Fuc*p* residues from XyG oligosaccharides [30]. However, the best *Arabidopsis* reciprocal BLAST match for the β-galactosidase was BGAL6/MUM3, an enzyme involved in removing terminal Gal*p* residues from pectin [31].

Figure 1 shows an RT-PCR analysis of the XyG-related contigs, confirming that these genes were also expressed in pollen tubes. Transcripts corresponding to the XyG-related contigs listed in Table 1 were amplified from pollen grain RNA and from the RNA isolated from pollen tubes grown in culture for 4, 8 and 16 hr. In each case, the sequenced amplicon matched the sequence of the contig in the transcriptome. To control for DNA contamination, mock reverse transcription reactions were performed such that reverse transcriptase was omitted from the reaction. In this case no transcripts were detected (data not shown). It is notable that all the XyG-related genes were also expressed in vegetative tissues such as leaves; indeed, in some instances such as the potential *MUR3* β-galactosyltransferase ortholog, the level of expression in pollen grains and tubes was noticeably lower than that in leaves. Consistent with previous observations *NaGSL1* was predominantly expressed in pollen grains and tubes with little expression in vegetative tissues. 

### Detection of XyG in *Nicotiana* pollen grain and pollen tubes

**Figure 2:**
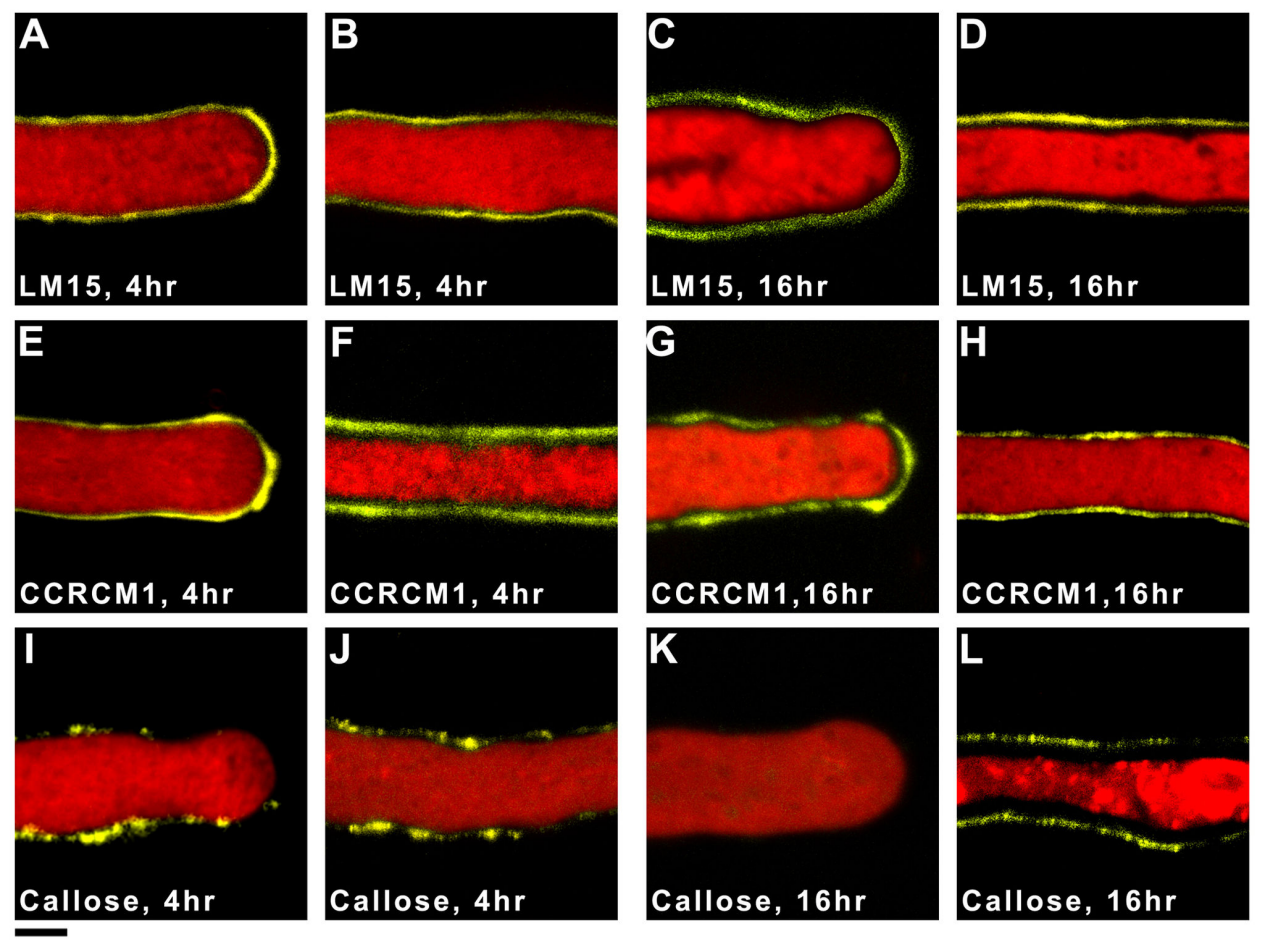
Immunofluorescence detection of XyG and callose in *Nicotiana* pollen tubes grown *in* *vitro* for 4 and 16 hr. Non-galactosylated and fucosylated XyG epitopes were detected (yellow) with the mAbs LM15 and CCRC-M1, respectively, and callose was detected with an anti-callose mAb. Pollen tubes were counter-stained with FM4-64 (red). A-D show 4 hr (A, B) and 16 hr (C, D) grown pollen tube tip (A, C) and shank (B, D) regions labelled with LM15: E-H show 4 hr (E, F) and 16 hr (G, H) tip (E, G) and shank (F, H) regions labelled with CCRC-M1: and I-L show 4 hr (I, J) and 16 hr (K, L) tip (I, K) and shank (J, L) regions labelled with the callose mAb. XyG epitopes were detected at the pollen tube tip and shank whereas callose epitopes were largely restricted to the shank region. Scale bar equals 5 µm.

The monoclonal antibodies (mAbs) LM15 and CCRC-M1 were used to detect XyG epitopes in *Nicotiana* pollen tubes grown in culture (Figure 2). LM15 binds to the XXXG motif of XyG and CCRC-M1 to the F (α-L-Fuc*p*-(1,2)-β-D-Gal*p*-(1-)) side chain (XyG oligosaccharide nomenclature as described in [32]). To provide contrast pollen tubes were counterstained with FM4-64 and the inner secondary wall defined using a mAb specific for callose, a known secondary wall polysaccharide in pollen tubes.

In pollen tubes grown for either 4 or 16 hr, LM15 labelling was evenly distributed along the entire length of the pollen tube including the pollen tube tip (Figure 2A-B, and C-D). Likewise, CCRC-M1 labelling was evenly distributed and covered the apex and shank of 4 hr (Figure 2 E-F) and 16 hr pollen tubes (Figure 2 G-H). The LM15 and CCRC-M1 labelling patterns were consistent with the presence of XyG epitopes in the primary wall and possibly also in the secondary wall of the pollen tube. By contrast, staining with the callose-specific mAb was patchy towards the tip and along the shank of 4 hr pollen tubes (Figure 2 I, J); by 16 hr labelling was absent from the apex but was still detectable along the shank (Figure 2 K, L). This labelling pattern was consistent with previous findings of callose in the secondary wall but not the pollen tube primary wall [4]. 

Finding XyG was likely being deposited at the pollen tube tip prompted us to re-examine the distribution of cellulose, as cellulose is frequently deposited in the wall along with XyG to form a cellulose/XyG network [33]. The dye Pontamine Fast Scarlet 4B (S4B), which fluoresces more brightly in the presence of cellulose than in the presence of XyG [34], was used to examine the distribution of cellulose in *Nicotiana* pollen tubes after 4 and 16 hr of growth. Figure S1 shows that S4B fluorescence was present at the apex of 4 hr pollen tubes but absent from the apex of 16 hr pollen tubes. In 16 hr pollen tubes, S4B staining was seen behind the tip but ahead of the point where aniline blue fluorochrome staining, which detects callose, was first evident. Thus, within the limits of this experiment, it appeared that *Nicotiana* pollen tubes deposit cellulose at their tip during the early stages of growth.

**Figure 3:**
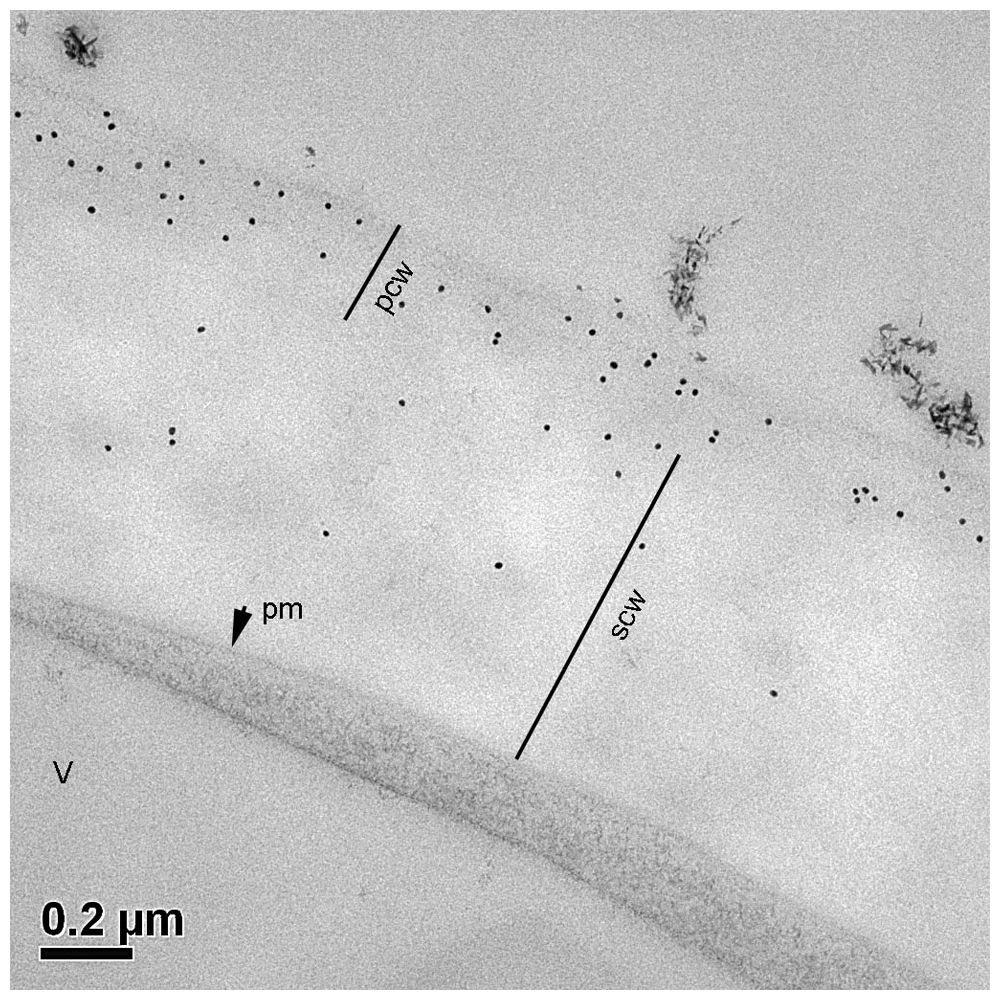
Immuno-gold transmission electron microscopy detection of XyG in *N. alata* pollen tubes grown in vitro for 16 hr. (A) Cross section labelled with mAb LM15 specific to the non-galactosylated (XXXG) motif of XyG. Labelling with gold particles (black dots) was predominantly in the outer, primary cell wall layer. pm=plasma membrane; pcw=primary cell wall; scw=secondary cell wall; v=vacuole.

Figure 3 is an immuno-gold transmission electron micrograph of LM15 labelling in a 16 hr pollen tube showing that XyG (specifically the XXXG structure of XyG) was present in the inner, electron-lucent secondary wall layer and the outer fibrillar primary wall layer of the bilayered tube wall, with the intensity of labelling in primary wall being higher than that in the secondary wall. 

### Structural analysis of XyG-derived oligosaccharides and chemical analysis of *Nicotiana* pollen grain and pollen tube walls

**Figure 4:**
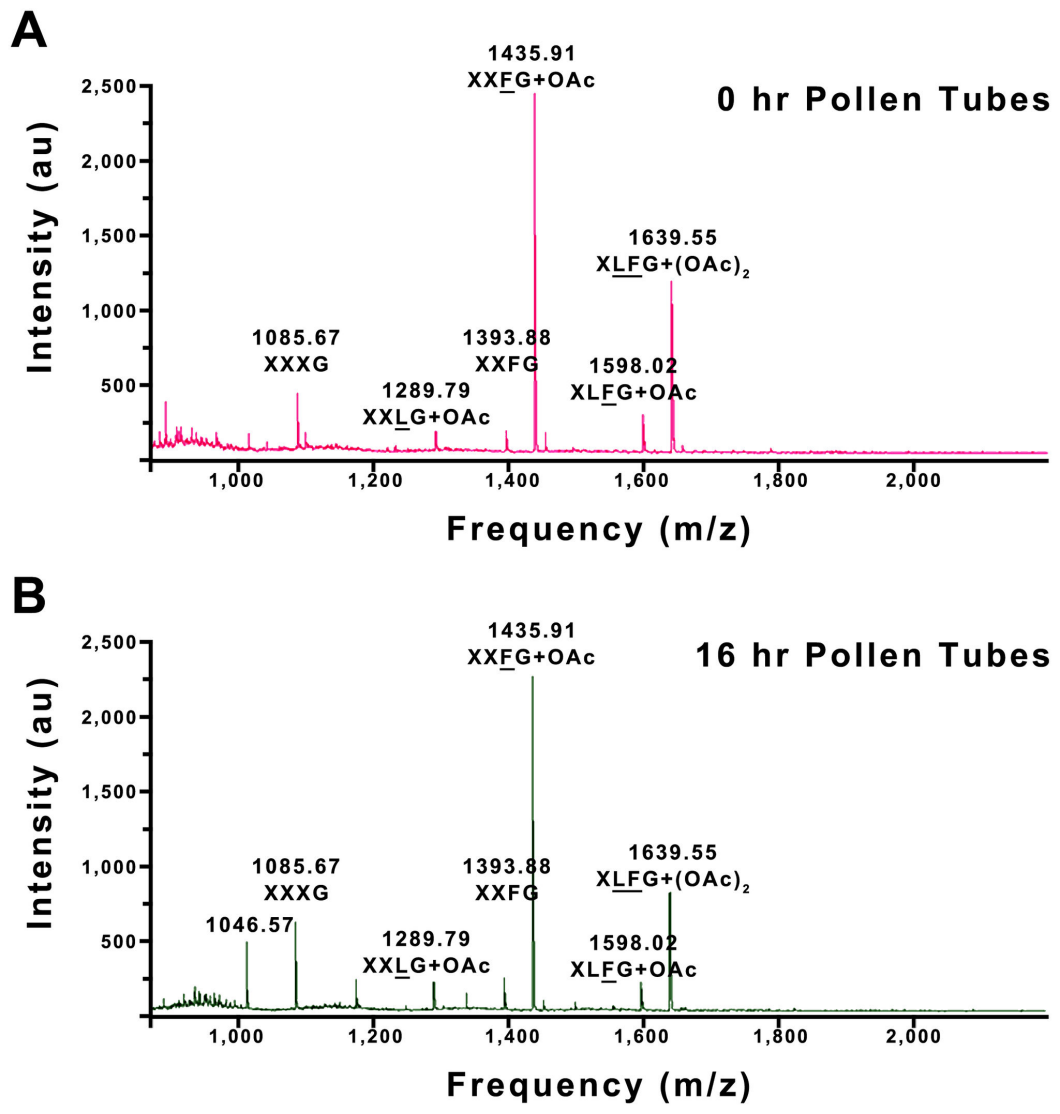
Analysis of *Nicotiana* pollen grain and pollen tube XyG. (A) shows MALDI-TOF MS analysis of endo-glucanase-generated XyG oligosaccharides released from pollen grain cell walls. (B) shows the XyG oligosaccharides released from 16 hr pollen tubes. XyG structures are annotated according to the nomenclature of Fry et al. [32] with underlined structures indicating the presence of an acetate ester group (OAc).

To further confirm the presence of XyG in *Nicotiana* pollen grain and pollen tube walls, matrix-assisted laser desorption ionization time-of-flight mass spectrometry (MALDI-TOF MS) analysis was performed on the cell wall oligosaccharides released after digestion with the XyG-specific endo-glucanase [21]. The MALDI-TOF MS profiles from pollen grains (Figure 4A) and 16 hr pollen tubes (Figure 4B) are diagnostic of vegetative dicot XyGs and are essentially similar, with two major (m/z 1435.91 and 1639.51; corresponding to XXFG + OAc and XLFG + (OAc)_2_, respectively) and four minor (m/z 1085.67, 1289.79, 1393.88 and 1598.02; corresponding to XXXG, XXLG + OAc, XXFG and XLFG + OAc, respectively) quasi-molecular ions. The glycosyl sequences, branching patterns and locations of the OAc groups of three oligosaccharides were verified by electrospray ionization tandem mass spectrometry (ESI-MS^n^, Figure S2). 

Linkages diagnostic of XyG were consistently present in methylation analyses of neutral carbohydrates in the walls of *Nicotiana* pollen grains and pollen tubes harvested at various time of growth in culture (Table 2). The other major neutral polysaccharides reported previously to be in *N. alata* pollen grains and pollen tube walls, cellulose and arabinan, were also found. Callose was not detected in pollen grains but levels rose rapidly after germination, indicating this polysaccharide is a major component of the pollen tube wall [6]. Low levels of linkages typical of the type II arabinogalactans (AGs), likely attached to proteins in the form of arabinogalactan proteins (AGPs), were also detected. This is consistent with the presence of AGPs reported in past immuno-localisation studies of *Nicotiana* pollen tubes [35,36].

**Table 2:** Glycosyl-linkage composition (mol%) of the neutral sugars in *N. alata* pollen-tube walls.

**Glycosyl residue**	**Deduced glycosyl linkage**	**Pollen-tube growth (hr)**							**Deduced polymer**
		**0**	**2**	**4**	**8**	**12**	**16**	**24**	
Rha*p*	2,4	1.2	1.1	1.2	1	0.9	0.7	0.8	other
Fuc*p*	terminal	1.4	1.6	1.2	1.4	2.9	0.5	1.6	XyG
Ara*f*	terminal	4.1	2.7	2.3	2.1	2.3	1.5	1.7	arabinan, AGP^1.^
	3-	1.5	1.1	0.9	0.9	0.9	0.3	0.8	arabinan
	5-	36.3	37	34.5	31	34.2	28.4	26.3	arabinan
	2,5-	1.5	2.8	2.8	3.1	3.4	2.4	2.9	arabinan
	3,5-	0.4	0.8	0.9	1.1	1.4	1.3	1.4	arabinan
Arap	terminal	4.1	2.7	2.3	2.1	2.3	1.5	1.7	AGP
Xyl*p*	2-	5.6	2.8	2.9	2	2.2	1.1	1.4	XyG
	4-	1.4	0.7	0.3	0.5	0.5	0.4	0.7	xylan
Gal*p*	terminal	2.6	2.1	1.8	1.5	1.4	1	1.2	XyG
	2-	4.4	1.7	1.8	1.4	1.6	0.2	1.2	XyG
	3-	0.9	1.7	1.5	0.6	0.4	1.2	0.3	AGP
	6-	1.8	4.1	3.8	3.3	2.9	2	2.1	AGP
	3,6-	2.1	1.8	1.4	0.9	0.7	0.7	0.5	AGP
Glc*p*	terminal	1	2.2	3.1	6.1	5.3	8.2	8.4	callose
	3-	0	5.6	9.8	16.8	12	24.5	24	callose
	4-	19	17	16.7	13.9	13.7	14.6	13.7	XyG, cellulose
	6-	0	0.1	0.1	0.2	0.2	0.1	0.2	unknown
	3,6	0	0.2	0.4	0.6	0.6	1.6	1	callose
	4,6	7	5.3	5.3	3.6	3.3	2.7	2.8	XyG
	2,3	0.1	0.2	0.3	0.4	0.5	0.5	0.6	callose

1 AGP = arabinogalactan protein.

Taken together, these data from the MALDI-TOF MS, methylation and immunofluorescence analyses indicate that XyG is present in the walls of *Nicotiana* pollen grains and is actively synthesised during pollen tube growth.

## Discussion

This paper describes how RNA-Seq analysis of *N. alata* pollen grains led to the discovery of fucosylated XyG in pollen tube walls and clearly demonstrates the advantages of RNA-Seq over previous approaches to gene profiling. The XyG-related transcripts in the transcriptome reported here do not appear among the over 413,000 *Nicotiana* ESTs currently available (as of June 2013) in GenBank or the over 43,000 unigenes present on the tobacco microarray [37]. Thus, even though the transcriptome was assembled from a single lane of sequence data, it identified many previously undescribed *Nicotiana* cDNAs.

*Nicotiana* pollen tube walls are reportedly composed mostly of callose, a (1,3)-β-glucan, with lesser amounts of cellulose and two pectic polysaccharides, an acidic homogalacturonan and a linear neutral (1,5)-α-arabinan, and some AGPs [5,6]. This composition indicated that *Nicotiana* pollen tube walls are unlike the primary walls that surround vegetative cells of dicots, which have a characteristic framework of cellulose microfibrils and non-covalently cross-linked xyloglucan embedded in a relatively porous matrix of pectic polysaccharides. Indeed, Li et al. [6] suggested XyG was essentially absent from *Nicotiana* pollen tube walls, with the trace amounts of XyG detectable by chemical analysis being derived from the pollen grain rather than from synthesis by the tube. This view must now change, as *Nicotiana* pollen tubes not only accumulate transcripts for proteins involved in XyG synthesis and remodelling, but also contain XyG that is predominantly but not exclusively located in the outer primary cell wall. Similarly, Dardelle et al. [38] recently reported XyG as a major component of both the inner and outer layers of *Arabidopsis* pollen-tube wall. In *Arabidopsis* pollen tubes, XyG is deposited at the tube apex along with highly methyl esterified homogalacturonan and cellulose, suggesting that the pollen tube primary wall in this species has a load-bearing cellulose-XyG framework as well as a pectic matrix, and thus resembles the primary walls that surround vegetative cells. 

The presence and location of microfibrillar cellulose in pollen-tube walls has been discussed many times in the past (e.g., [39]). Based on an electron microscopy study using gold-labelled cellobiohydrolase (CBH1), Ferguson et al. [4] concluded that cellulose was not in the primary wall and tube tip of *Nicotiana* pollen tubes but was co-located with callose in the inner secondary wall. Derksen et al. [2], however, recently found long fibres that were presumed to be composed of cellulose in a region of primary wall behind the tip. Similarly, Cai et al. [40] used an anti-CESA antibody to detect cellulose synthases in *Nicotiana* pollen tubes, and found the highest level of protein labelling was at the pollen tube tip. Although this antibody was most likely not detecting a pollen tube CESA but the closely related CSLDs, as CSLDs are suggested to be the cellulose synthases in *Nicotiana* pollen [7], this observation raised the possibility of cellulose being deposited in the primary wall at the tip as well as in the secondary wall. The staining pattern obtained with pontamine fast scarlet 4B (S4B) is consistent with this interpretation, at least in the early stages of growth. Notably, although S4B also binds weakly to XyG and callose [34], differences in the staining patterns of XyG and callose (Figure 4) and S4B (Figure S1) in 16 hr pollen tubes in particular suggest that the other two polysaccharides were not labelled to a significant extent by S4B. Thus it appears likely that there is cellulose at the *Nicotiana* pollen tube tip, and hence possibly a cellulose/XyG network as well, during the first few hours of growth. As S4B staining at the apex of 4 hr pollen tubes was strong, it is further possible that cellulose synthesis is not restricted to the plasma membrane but also occurs in vesicles ahead of surface deposition, as has been reported in *Arabidopsis* pollen tubes [41]. In older pollen tubes cellulose was not present at the apex but was seen behind the tip ahead of the place where callose is deposited, an observation consistent with a previous description of cellulose deposition in *Nicotiana* pollen tubes [4]. 

XyGs have a linear backbone of (1,4)-linked β-Glc residues with up to 75% of residues substituted at C(O)6 by Xyl to form a core XXXG structure (nomenclature of [32]). Further substitution of Xyl residues in this core builds up the di- and tri-glycosyl side branches typical of XyGs like the XXFG and XLFG structures seen in *Nicotiana* pollen grains and pollen tubes (Figure 4). The presence of fucosylated XyGs in *Nicotiana* pollen grains and tubes was consistent with the immunofluorescence labelling pattern seen with the monoclonal antibody CCRC-M1, which binds terminal Fuc residues linked α-(1,2)- to a galactosyl residue, the epitope found in the F side chain of XyG [42,43] and with other studies of *Arabidopsis* pollen grains and pollen tubes where fucosylated XyGs are also found [38,43]. However, although XXFG and XLFG are XyG structures found in many dicot cell walls (e.g., see 44), fucoslyated XyGs are not expected in a solanaceous plant like *Nicotiana*, where previous studies of the XyGs in vegetative tissues have found less Xyl substitution (a XXGG core structure instead of the more usual XXXG) and no Fuc residues [45,46]. Instead of Fuc, the XyG side chains in the Solanaceae have Ara residues attached to some of the Xyl [45]. The major XyG structures expected in *Nicotiana* are thus XSGG (where S is Ara-(1,3) Xyl) and XXGG [10,47], neither of which was found in pollen grains and tubes (Figure 4). 

It is difficult to understand the need for such dissimilar XyG structures in the walls surrounding *Nicotiana* vegetative cells and pollen grains and tubes. One possibility is that it relates to the different functional requirements for growth of these two cell types: XyG supposedly has a major role in cell growth [33] and there are obvious differences between pollen tubes and vegetative cells in their manner of growth (tip-focussed versus diffuse). For this possibility to be true, however, changes in growth would need to accompany changes to the fine structure of XyG. But the picture that has emerged recently has challenged models of the functional organisation of primary cell walls by showing that growth is largely unaffected by changes to XyG structure. For example, although the XyG made by the *Arabidopsis mur1* mutant has less than 2% of the wild-type level of Fuc, no phenotypic consequences are attributed to this change [48,49]. Similarly severe changes in XyG structure are reported for the *Arabidopsis mur2* and *mur3* mutants yet these plants too appear phenotypically normal [28,50]. Even *Arabidopsis* mutants with no detectable XyG display only relatively minor phenotypic consequences as a result of the loss, presumably because other wall polysaccharides assume the load-bearing functions of the missing XyG [51-53]. Thus, while the function of XyG in tip-growing cells has not been extensively studied, there is currently no reason to believe that producing fucoslyated XyGs is a mechanically important feature of the wall and plays a key role in the growth of tobacco pollen tubes. The availability of the XyG-related gene sequences described here does, however, allow this possibility to be experimentally tested. More interestingly, these sequences can be used to explore XyG synthesis in *Nicotiana* vegetative tissues, especially as it appears most of the genes needed to make the XXFG and XLFG structures seen in pollen grains are also expressed in leaves (Figure 1).

As already noted, previous studies of *Nicotiana* pollen tube have found the biochemical composition of the cell wall changes from tip to shank. This study has confirmed the existence of one of these gradients (for callose) but found evidence that another gradient (for cellulose) may be dependent on growth stage, as S4B fluorescence in 4 hr pollen tubes was at the apex but was behind the apex in 16 hr pollen tubes. There have been several recent attempts to model the elongation of tip-growing cells such as pollen tubes (see 54). Those models are based on growth of a walled cell and often assume that the mechanical properties of the wall remain constant over time, with wall assembly more or less focused at the apex and/or in a region slightly behind the growing point (e.g., see 55,56). Temporal changes to the biochemical composition of the wall have not been considered but may need to be incorporated in future experimental studies and models of tip growth.

## Supporting Information

Table S1 **Summary statistics for the *N. alata* pollen grain transcriptome.**
(PDF)

Table S2 **Validation of the *N. alata* pollen grain transcriptome.**
(PDF)

Table S3 **List of DNA primers used in this study.**
(PDF)

Figure S1 **Fluorescence detection of cellulose (as detected by S4B staining) and callose (as detected by aniline blue fluorochrome staining) in *N. alata* pollen tubes.** Scale bar equals 5 µm.
(PDF)

Figure S2 **ESI-MS/MS analysis of XyG oligosaccharides released by endoglucanase treatment of *Nicotiana* pollen grains.**
(PDF)
