# Supplementary material for: In Vitro Grown Pollen Tubes of Nicotiana alata Actively Synthesise a Fucosylated Xyloglucan
Source: PLoS One. 2013 Oct 8;8(10):e77140. doi: 10.1371/journal.pone.0077140 (PMC3792914; doi:10.1371/journal.pone.0077140)
Supplement: Table S1 — Summary statistics for the N. alata pollen grain transcriptome. (PDF) [file pone.0077140.s001.pdf]

Supplemental Table 1: Summary statistics for the *N. alata* pollen grain transcriptome.

***N. alata* transcriptome statistical summary**	
Total number of 75 base pair reads	7,698,092
Total number of contigs generated	11,049
Total transcriptome length (bp)	5,828,264
Average contig length (bp)	528
Median contig length (bp)	341
Range in length (bp)	201-6,983
L50[1.]	682
N50[2.]	2,354

***N. alata* reads mapped to *N. benthamina* genomic[3.]**	
overall alignment	7,623,374 (90.36%)
aligned 0 times	741,718 (9.64%)
aligned exactly 1 time	4,853,457 (63.05%)
aligned >1 time	2,102,928 (27.32%)

[1.] L50 is the length of the contig that separates the top half (N50) of the assembled transcriptome from the remainder of smaller contigs, if the sequences are ordered by size from smallest to longest.

[2.] N50 is the number of contigs that represent the top half of the transcriptome, if the sequences are ordered by size from smallest to largest.

[3.] The *N. benthamina* genomic draft sequence version 0.4.2 was used in this analysis (Bombarely et al. 2012).
