# Supplementary material for: In Vitro Grown Pollen Tubes of Nicotiana alata Actively Synthesise a Fucosylated Xyloglucan
Source: PLoS One. 2013 Oct 8;8(10):e77140. doi: 10.1371/journal.pone.0077140 (PMC3792914; doi:10.1371/journal.pone.0077140)
Supplement: Table S2 — Validation of the N. alata pollen grain transcriptome. (PDF) [file pone.0077140.s002.pdf]

Supplemental Table 2: List of 56 known pollen-expressed genes from tobacco (*N. alata* and *N. tabacum*) used for validation of the *N. alata* pollen grain transcriptome. The indicated sequence was used to query the 11,049 contigs over 200 bp in length in the pollen grain transcriptome. The top BLAST hit for each gene is shown along with the contig's length and pairwise identity to the query.

Query			Contig				
**gene name**	**accession No.**	**comments**	**ID number**	**DDBJ accession No.**	**length (bp)**	**pairwise identity (%)**	**RPKM**
N. alata							
DD1	EF420251.1	F-box protein	-		-	-	-
DD2	EF420252.1	"	6442	AB844164	362	98.3	26
			6581	AB844166	433	97.2	24
DD3	EF420253.1	"	3818	AB844158	281	99.3	31
DD4	EF420254.1	"	2664	AB844153	1357	99.6	40
DD5	EF420255.1	"	-		-	-	-
DD6	EF420256.1	"	-		-	-	-
DD7	EF420257.1	"	-		-	-	-
DD8	EF420258.1	"	-		-	-	-
DD9	EF420259.1	"	6464	AB844165	237	99.6	26
			7866	AB844169	473	100	17
DD10	EF420260.1	"	-		-	-	-
GSL1	AF304372.2	Putative callose synthase	290	AB844131	6983	100	854
CSLD1	AF304375.1	Cellulose synthase D-like	567	AB844140	2183	99.6	265
CESA1	AF304374.1	Cellulose synthase	-		-	-	-
P18	AJ004957.1	Hypothetical protein	104	AB844126	981	99.8	3,473
SBP1	EU591514.1	RING domain protein	-		-	-	-
MIP	U20490.1	Probable aquaporin	92	AB844125	875	99.6	1,875
PCCP	EU591515.1	C2 domain containing protein	65	AB844123	977	99.8	4,007
N. tabacum							
ADF1	AY081941.1	Actin-depolymerizing factor	-		-	-	-
ADF2	AY081942.1	"	408	AB844135	236	96	688
RHOGD2	DQ416769.1	Rho GDP-dissociation inhibitor	171	AB844128	1413	96.2	1,640
Nict1	AB035706.1	Calcium binding protein	127	AB844127	711	95.2	2,197
Nict2	AB035705.1	"	2352	AB844151	764	92.5	31
CysP	EU429306.1	Cysteine protease	-		-	-	-
NTP805	AY366400.1	Pollen-specific protein	71	AB844124	370	96.1	2,524
PNTP302	AY366399.1	"	-		-	-	-
NTP303	X61146.1	"	15	AB844118	2052	94.1	8,760
PLIM1	AF184885.1	LIM domain-containing protein	63	AB844122	1064	98	4,558
PLIM2	AF116851.1	"	-		-	-	-
AscOx	X96932.1	Ascorbate oxidase	-		-	-	-
PLC3	EF043044.1	Phospholipase C	569	AB844141	2102	95.5	174
SuSy	EU148354.1	Sucrose synthase	-		-	-	-
PRK1	AF246964.1	Receptor-like protein kinase	744	AB844143	1332	97.4	354
			474	AB844136	1205	95.8	486
PRK2	AF246967.1	"	334	AB844132	2453	97.3	811
PRK4	AF252414.1	"	564	AB844139	2233	95	335
GNL1	EF520731.1	GNOM-like protein	1128	AB844146	3450	97.6	76
eIF-4A	X79005.1	Translation initiation factor	-		-	-	-
NPG1	X71020.1	Polygalacturonase	55	AB844121	1686	97.7	6,452
PPME	AY772945.1	Pectin esterase	4	AB844117	2061	95.3	37,610
NHA1	AY383599.2	H^+ ATPase	29	AB844119	2371	98.2	7,402
AldH 2A	Y09876.1	Aldehyde dehydrogenase	5713	AB844162	343	98	30
			6850	AB844167	247	97.1	24
			5427	AB844161	204	93.3	26
PDC2	X81855.1	Pyruvate decarboxylase	6069	AB844163	392	98.4	25
NTK-1	X77763.1	Shaggy-like kinase	-		-	-	-
PL	X67159.1	Pectate lyase	-		-	-	-
ROP1	AJ222545.2	Rop subfamily GTPase	989	AB844145	409	97.1	222
NSK 91	AJ224163.1	Shaggy-like kinase	346	AB844133	1520	95.3	315
NSK 59	AJ002315.1	"	785	AB844144	552	96.4	288
NSK 111	AJ002314.1	"	644	AB844142	295	99	544
Rac1	AY029330.1	Rac-like GTPase	187	AB844129	574	95.8	688
PK2	AJ608157.1	Ser/Thr protein kinase	536	AB844138	725	95.9	142
PK1	AJ608156.1	"	1216	AB844147	304	95.4	212
			1236	AB844148	552	92.3	165
TP5	AJ250431.1	Putative β-galactosidase	40	AB844120	2869	95.8	7,212
RAB2	AF397451.1	Rab2 GTPase	1596	AB844149	318	100	115
mybAS1	AF198499.1	Myb-related protein	4402	AB844160	268	91.7	38
mybAS2	AF198498.1	"	2517	AB844152	917	96.1	53
			2833	AB844154	372	96.9	56
JD1	AF316320.1	Putative Ca^{2+}-binding protein Sucrose transporter protein	477	AB844137	1770	96.1	258
SUT3	AF149981.1	Sucrose transporter-like protein	399	AB844134	2103	94.9	281
