# Supplementary material for: In Vitro Grown Pollen Tubes of Nicotiana alata Actively Synthesise a Fucosylated Xyloglucan
Source: PLoS One. 2013 Oct 8;8(10):e77140. doi: 10.1371/journal.pone.0077140 (PMC3792914; doi:10.1371/journal.pone.0077140)
Supplement: Table S3 — List of DNA primers used in this study. (PDF) [file pone.0077140.s003.pdf]

Supplemental Table 3. DNA Primers

Locus	Predicted function	Abbreviation	Number of hits	Contig ID (DDBJ Accession No.)	Primer name	Primer sequence	Product size (bp)
At2g03220	(1,2) -α-L-fucosyltransferase	*At*FUT1	0				
At2g20370	(1-2)-β-galactosyltransferase	*At*KAM3/ MUR3	1	6942 (AB844168)	Na6942-01F	ATGCCGAGATTGGAACCTTT	278
					Na6942-02R	TTCGTCGTAAACCAACCTTTG	
At3g28180	(1-4)-β-D-glucan synthase	*At*CSLC4	1	3421 (AB844156)	Na3421-01F	TGATCTTGAGGCTGGTGATG	1106
					Na3421-02R	AAGGTATGGCACGACGAAAG	
					Na3421-03F	CACGGGTGGAAATTCATCTT	287
					Na3421-04R	GTCGGGAGTGTAGCTTCAGG	
At4g07960	(1-4)-β-D-glucan synthase	*At*CSLC12	1	8368 (AB844170)	Na8368-01F	TGGAAAATCCAAATAATTGGTCA	203
					Na8368-02R	GCTGATGCTATGGAAGTTAAACAA	
At1g74380	(1-6)-α-D-xylosyltransferase	*At*XXT5	2	2900 (AB844155)	Na2900-01F	GCTTTTGCTTTCACATCCTGA	720
					Na2900-02R	GCTTTAAACGGTGGGTGAAG	
					Na2900-03F	CAGAAGGAGAAATGGGGTGA	331
					Na2900-04R	CTTCAAGCGGATTGCTTGTT	
				4256 (AB844159)	Na4256-01F	ATCGGTTCTTGAGTGCTCGT	411
					Na4256-02R	TCAACCACTCACCTCTTTGCT	
					Na4256-03F	GGGTGCTGGAAAATTTGGTA	291
					Na4256-04R	CCTCTTTGCTCATCCCAATC	
At5g57550	endo-(1, 4)-β-D-glucanase	*At*XTH25	1	3443 (AB844157)	Na3443-01F	GGAAATTCAGCTGGCACTGT	609
					Na3443-02R	CGCAGGTCCTTTAGGAAACC	
					Na3443-03F	TGCAGATGATTGGGCTACAC	271
At1g32170	endo-(1, 4)-β-D-glucanase	*At*XTH30	1	1953 (AB844150)	Na1953-01F	TCTTTGACACCCTTCTCTCTCC	920
					Na1953-02R	TCTTCTGCCTAAGCCTTCCA	
					Na1953-03F	GGATGCATCTTCTTGGGCTA	324
At1g67830	(1-2)-α-L-fucosidase	*At*FXG1	1	263 (AB844130)	Na263-01F	AGCGGAGCAAAGTACATCGT	297
					Na263-02R	TCCTGTAGCTCCACAGCAAG	
At2g13680	(1-3)-β-D-glucan synthase	*At*GSL2	1	290 (AB844131)	Na290-01F	CATCATGAACCTGCAGTTGG	955
					Na290-02R	AAAAGCAGCCTTGTCTGGAA	
					Na290-03F	GTGGCTCAATGTGGTTCCTT	322
					Na290-04R	CGAACACAATGACCAACCAG	
