# Supplementary material for: In Vitro Grown Pollen Tubes of Nicotiana alata Actively Synthesise a Fucosylated Xyloglucan
Source: PLoS One. 2013 Oct 8;8(10):e77140. doi: 10.1371/journal.pone.0077140 (PMC3792914; doi:10.1371/journal.pone.0077140)
Supplement: Figure S1 — Fluorescence detection of cellulose (as detected by S4B staining) and callose (as detected by aniline blue fluorochrome staining) in N. alata pollen tubes. Scale bar equals 5 µm. (PDF) [file pone.0077140.s004.pdf]

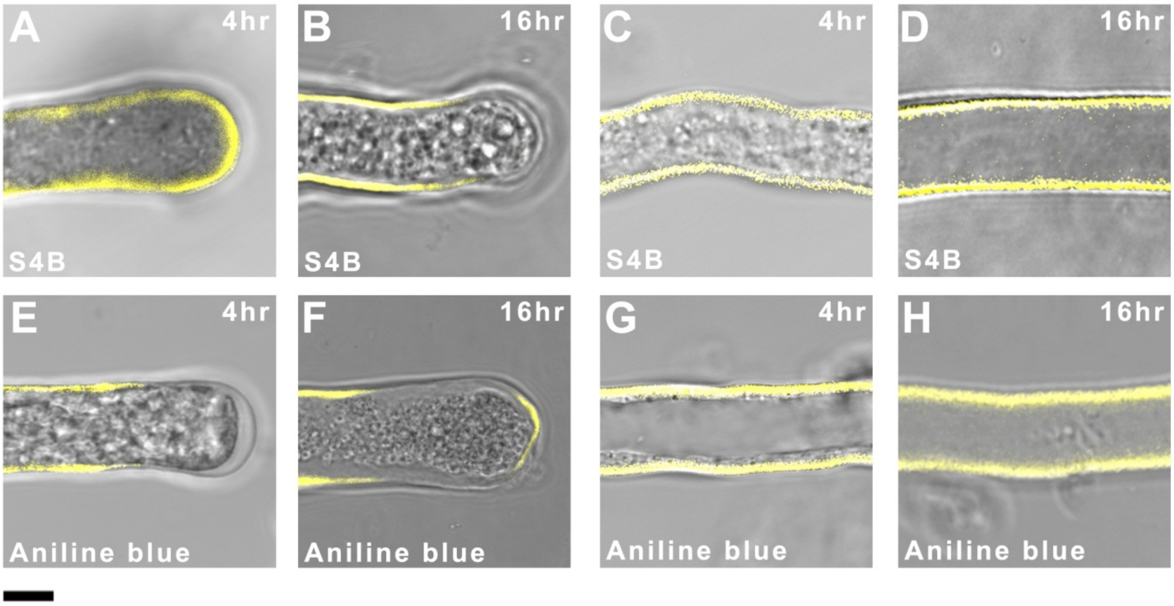

Supplemental Figure 1: Fluorescence detection of cellulose (S4B; A-D) and callose (aniline blue; E-H) in the tip (A, B, E, F) and shank (C, D, G, H) regions of 4 hr and 16 hr *N. alata* pollen tubes. Scale bar equals 5 µm.
