# Supplementary material for: In Vitro Grown Pollen Tubes of Nicotiana alata Actively Synthesise a Fucosylated Xyloglucan
Source: PLoS One. 2013 Oct 8;8(10):e77140. doi: 10.1371/journal.pone.0077140 (PMC3792914; doi:10.1371/journal.pone.0077140)
Supplement: Figure S2 — ESI-MS/MS analysis of XyG oligosaccharides released by endoglucanase treatment of Nicotiana pollen grains. (PDF) [file pone.0077140.s005.pdf]

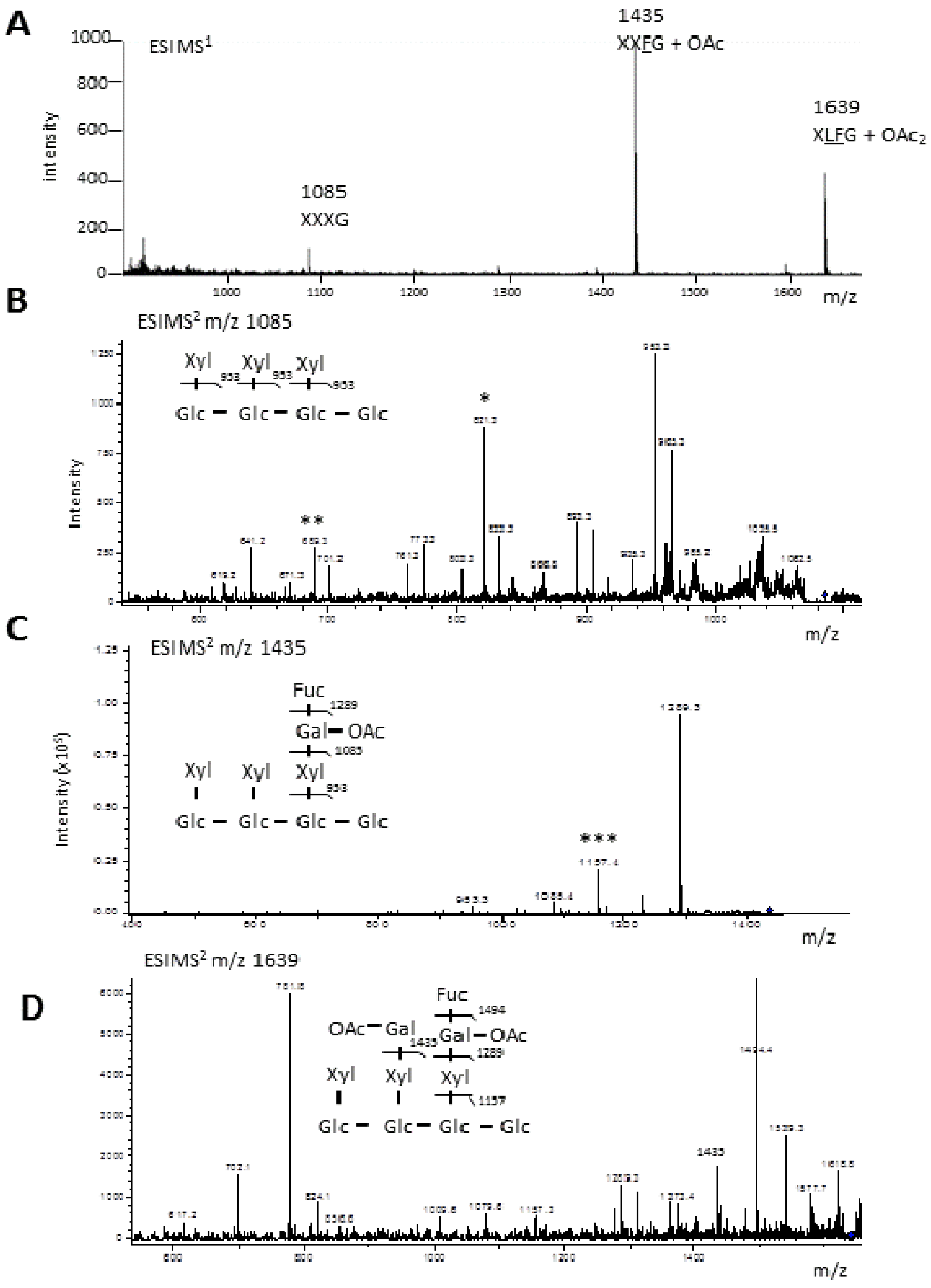

Supplemental Figure 2: The signals of ESI-MS spectra of XyG oligosaccharides released by XyG-specific endoglucanase treatment of 16 hr *Nicotiana* pollen tubes are identified as $[M+Na]^+$ ions. A: The quasi-molecular ions observed in the ESI-MS1 spectrum. B: The ESIMS2 spectrum recorded upon the fragmentation of the quasi-molecular ions at m/z 1085 consists of ion at m/z 953 generated by the loss of one non-reducing xylosyl residue. Further loss of one or two non-reducing xylosyl residues generated the ions m/z 821* and 689 **, respectively. C: The ESIMS2 spectrum recorded upon the fragmentation of the quasi-molecular ions at m/z 1435 includes the most abundant fragment ion at m/z 1289 generated by loss of terminal fucosyl residue. Loss of both terminal fucosyl residue and a terminal xylosyl residue generated the product ion m/z 1157***. D: The ESIMS2 spectrum recorded upon fragmentation of the quasi-molecular ions at m/z 1639.
